# Perirectal hematoma after stapled surgery for hemorrhoidal prolapse and obstructed defecation syndrome: case series management to avoid panic-guided treatment

**DOI:** 10.1007/s13304-023-01490-y

**Published:** 2023-03-10

**Authors:** Domenico Mascagni, Chiara Eberspacher, Gabriele Naldini, Francesco Leone Arcieri, Pietro Mascagni, Roberto Cirocchi, Georgi Popivanov, Pierpaolo Sileri, Stefano Arcieri

**Affiliations:** 1 Department of Surgery, University of Rome “Sapienza”, Viale Regina Elena 324, 00100 Rome, Italy; 2 Proctology and Pelvic Floor Clinical Centre, Cisanello University Hospital, Pisa, Italy; 3 Fondazione Policlinico Universitario Agostino Gemelli IRCCS, Rome, Italy; 4 Department of General Surgery, University of Perugia, 06100 Perugia, Italy; 5 Department of Surgery, Military Medical Academy, 1606 Sofia, Bulgaria; 6 University Vita-Salute San Raffaele, Milan, Italy

**Keywords:** Perirectal hematoma, Stapler, Hemorrhoids, Complications

## Abstract

Perirectal hematoma (PH) is one of the most feared complications of stapling procedures. Literature reviews have reported only a few works on PH, most of them describing isolated treatment approaches and severe outcomes. The aim of this study was to analyze a homogenous case series of PH and to define a treatment algorithm for huge postoperative PHs. A retrospective analysis of a prospective database of three high-volume proctology units was performed between 2008 and 2018, and all PH cases were analyzed. In all, 3058 patients underwent stapling procedures for hemorrhoidal disease or obstructed defecation syndrome with internal prolapse. Among these, 14 (0.46%) large PH cases were reported, and 12 of these hematomas were stable and treated conservatively (antibiotics and CT/laboratory test monitoring); most of them were resolved with spontaneous drainage. Two patients with progressive PH (signs of active bleeding and peritonism) were submitted to CT and arteriography to evaluate the source of bleeding, which was subsequently closed by embolization. This approach helped ensure that no patients with PH were referred for major abdominal surgery. Most PH cases are stable and treatable with a conservative approach, evolving with self-drainage. Progressive hematomas are rare and should undergo angiography with embolization to minimize the possibility of major surgery and severe complications.

## Introduction

In the last 20 years, the proctological surgery panorama has seen a large application of stapler-related techniques for patients affected by hemorrhoidal prolapse or obstructed defecation syndrome (ODS) with rectocele and intussusception [1–5].

The use of surgical staplers is safe and offers remarkable levels of satisfaction among both patients and surgeons [6]. For patients with hemorrhoidal prolapse, stapling guarantees a decrease in postoperative pain and a faster return to daily activities when the technique is performed by skillful operators and with the correct indications [7, 8]. Moreover, a significant improvement in defecatory function has been detected in ODS patients operated on with this technique [5–7].

Stapling techniques, similar to any proctological surgical procedure, can be burdened by perioperative and postoperative complications [9–12]. Some of these complications are common and easy to treat, such as pain, bleeding, and recurrence. Rare but undoubtedly severe complications may been reported after stapler surgery, including large anastomotic dehiscence with pelvic sepsis, rectal ischemia/necrosis, rectal perforation with pelvic peritonitis, hemoperitoneum/pneumoperitoneum, rectovaginal fistula and perirectal hematoma (PH) [13–22].

The present work involved a significant case series of PH among a large cohort of 3058 patients who underwent stapler surgery in three high-volume proctologic units. The aim of this study was to analyze the evolution and management of these cases to define a treatment algorithm for huge postoperative PH cases.

## Methods

In this case series, a retrospective analysis of a prospective database of three high-volume proctology units was conducted between 2008 and 2018. All cases of PH occurred after stapling procedures for hemorrhoidal disease or ODS with internal prolapse. PH refers to severe extra-visceral bleeding associated with the development of massive, symptomatic blood collection in the pelvis around the rectum.

Diagnostic workup, treatment and clinical evolution were considered for each case in the study. A collaboration between the three proctologic units (“Sapienza” University of Rome; University of Rome “Tor Vergata”; Cisanello University Hospital Pisa) resulted in a high volume of patients who underwent stapled procedures, which minimized the risk of bias resulting from poor surgical experience with this procedure. All surgeons had performed at least 100 stapled operations for hemorrhoids or ODS before the lapse of the time of evaluation. Surgeries were performed according to the same technical guidelines.

Patients who underwent stapled transanal rectal resection (STARR) for ODS treatment were previously informed about the other surgical procedures available, namely transanal and transabdominal techniques, after the failure of nonsurgical approaches (diet, medical therapy and rehabilitation). The decision to conduct the STARR procedure was made by the surgeon together with the informed patients. 61% of the initial cohort of patients included in the study were women, and the average age was 57 years; 6% of all patients had a history of anal surgery for proctologic diseases, but all cases with a recurrence of prolapse were excluded to minimize bias.

## Results

Between January 2008 and December 2018, 3058 patients underwent stapling procedures for hemorrhoidal disease or ODS with internal prolapse, and 14 of these patients were reported to have large PHs (0.46%) (Table 1). All 14 patients were female, aged 41–70 years (mean age: 56 years). None of these patients had undergone proctological or gynecological surgery prior to the study period, and they were not on any antiplatelet or anticoagulant medication. Among them, ten patients underwent stapled procedures for internal rectal prolapse and rectocele with ODS and the remaining four for hemorrhoidal prolapse. Different types of staplers and surgical techniques were used: two patients underwent hemorrhoidopexy (one with PPH01, one with PPH03), five underwent STARR with two staplers (two PPH01 in three cases and two PPH03 in two cases), and seven underwent STARR with one high-volume stapler (one CPH 34, three CPH36 and three TST 36).

**Table 1:** Baseline characteristics of 14 patients with postoperative hematoma in the database of 3058 patients who underwent stapling procedures between 2008 and 2018

Group	Pts	Sex	Age	Indication for surgery	Surgical technique (device)	Time of onset (PO day)	Rectal bleeding	Other symptoms
I	1	F	66	Prolapse and rectocele	STARR HV CPH36	II	Yes	Anal pain
I	2	F	50	Prolapse and rectocele	STARR 2 PPH01	III	Yes	Anal pain, Peritonism (urinary retention)
I	3	F	56	Prolapse and rectocele	STARR 2 PPH03	V (post dismission)	No	Anal pain
I	4	F	57	Prolapse and rectocele	STARR HV CPH36	Immediate	Yes	Anal pain
I	5	F	59	Prolapse and rectocele	STARR HV CPH36	I	No	Peritonism (urinary retention)
II	6	F	55	Prolapse and rectocele	STARR 2 PPH01	VII (post dismission)	No	Anal pain, fever
II	7	F	41	Hemorrhoidal prolapse	PPH01	Immediate	No	Anal pain, Peritonism (urinary retention)
II	8	F	62	Prolapse and rectocele	STARR HV TST36	Immediate	No	Anal pain, Peritonism (urinary retention)
II	9	F	60	Hemorrhoidal prolapse	STARR HV CPH34	Intraop.	Yes	Anal pain, Bleeding
III	10	F	42	Hemorrhoidal prolapse	STARR 2 PPH01	Immediate	No	Anal pain
III	11	F	56	Prolapse and rectocele	STARR 2 PPH03	Immediate	Yes	Anal pain
III	12	F	70	Hemorrhoidal prolapse	1 PPH03	Immediate	Yes	Anal pain
III	13	F	61	Prolapse and rectocele	STARR HV TST36	I	No	Anal pain, Peritonism (urinary retention)
III	14	F	54	Prolapse and rectocele	STARR HV TST36	Immediate,progressive	No	Anal pain, Peritonism (urinary retention)

PH onset was intraoperative in one case, immediately postoperative (PO) in seven cases, within the first three PO days during hospitalization in four cases, and after hospital discharge in two cases (one was readmitted for evaluation and therapy; the other was clinically stable and followed up on an outpatient basis). The main symptoms of PH were anal pain (in all cases) and postoperative peritonism (six cases, 43%). Notably, no rectal bleeding was observed in eight out of 14 patients (57%). In one case, the hematoma was identified intraoperatively and subsequently associated with postoperative anal pain and bleeding.

The first diagnostic step was digital rectal examination, which raised a suspicion of PH in all 14 patients. A pelvic contrast CT scan was performed in all cases, whereas an endoluminal ultrasound was carried out in five cases. PH size ranged from 35 × 40 cm to 90 × 75 cm. Regarding anatomical localization, the following patterns were observed: a mainly posterior location in 10 cases, a mainly anterior site in two cases, and a circumferential location in two cases.

All 14 patients were administered IV fluids and an antibiotic treatment (association of metronidazole and ciprofloxacin) (Table 2). An evaluation of serial full blood count and inflammation markers (C-reactive protein and procalcitonin) was performed for all patients. Four patients required blood transfusions. In six cases with abdominal tenderness, a bladder catheter was inserted.

**Table 2:** Diagnosis and treatment of 14 patients with PH after stapling procedures

Groups	Pts	Imaging	Location	Volume (mm)	Monitoring, medical therapy	2° surgery evolution	Dismission (PO day)	Follow- up (1 year)
I	1	DE, EUS, CT, RM	Posterior	55 × 85	Blood test/Antibiotics/Fluids/EUS/ CT	Spontaneous evacuation (X)	XI	Ok
I	2	DE, EUS, CT	Posterior	35 × 45	Blood test/Antibiotics/Fluids/NPT/Urinary catheter/EUA	EUA/Spontaneous evacuation (XIII)	XIV	Ok
I	3	DE, CT	Posterior	55 × 70	Blood test/Antibiotics/Fluids/TC/Blood transfusion	Dehiscence/Spontaneous evacuation/EUA/Reinforcement of anastomosis (IV)	VII	Ok
I	4	DE, CT	Anterior	55 × 60	Blood test/Antibiotics/Fluids/CT	EUA Spontaneous evacuation (V)	VII	Ok
I	5	DE, CT, RM	Posterior	70 × 85	Blood test/Antibiotics/Fluids/NPT/Transfusion/ Urinary catheter/CT	Spontaneous evacuation EUA/Reinforcement anastomosis (VII)	IX	Ok
II	6	DE, CT	Posterior	70 × 60	Blood test/Antibiotics/CT	No surgery/ Chronic disappeared (XVI)	No admission	Ok
II	7	DE, EUS, CT	Posterior	80 × 70	Blood test/Antibiotics/Fluids/Urinary catheter/EUA/CT with drainage	EUA/Surgical drainage /CT drainage/Chronic sdisappered (XXX)	VII	OK
II	8	DE, CT	Posterior	50 × 40	Blood test/Antibiotics/Fluids/Urinary Catheter/ CT			
II	9	DE, CT	Anterior	35 × 40	EUA-Intraoperative stitches/Blood test/Antibiotics/Fluids/CT	EUA- Surgical Drainage (III) Chronic disappeared (XV)	V	Ok
III	10	DE, CT	Circumferential	50 × 40	Blood test/Antibiotics/Fluids/CT	EUA Spontaneous evacuation (VIII)	IX	Ok
III	11	DE, EUS, CT	Posterior	35 × 40	Blood test/Antibiotics/Fluids/CT	Spontaneous evacuation (VI)	VII	Ok
III	12	DE, CT	Circumferential	60 × 50	Blood test/Antibiotics/Fluids/CT	Spontaneous evacuation (IV)	V	Ok
III	13	DE, CT, Arteriography	Posterior	80 × 70	Blood test/Antibiotics/Fluids/NPT/Urinary catheter/CT/Blood transfusion/Arteriography	Arteriography/Chronic disappeared (XV)	VI	Ok
III	14	DE, CT, Arteriography	Posterior	90 × 75	Blood test/Antibiotics/Fluids/NPT Bladder catheter/CTt/Blood transfusion/Arteriography	Arteriography/Chronic Diappeared (XII)	IV	Ok

Two of these six patients complained of sudden hypotension. Immediate resuscitation and hemodynamic stabilization procedures were undertaken. A prompt angio-CT scan and subsequent arteriography were performed for these patients to evaluate the PHs and presence of bleeding, with the active sources closed via embolization.

No patients were referred for abdominal surgery.

Eight cases were referred for anorectal evaluation under anesthesia. Transanal partial drainage of PH was achieved in one case, while it was ineffective in the other three cases; in two cases of anal bleeding, hemostasis was performed with transparietal stitches. After spontaneous drainage, the anastomotic leak was transanally repaired with perianastomotic stiches in two patients.

Transanal spontaneous drainage of PH occurred in nine cases. One patient with late-onset hematoma was not readmitted to the hospital; three patients were discharged before Day 5, while nine patients were discharged between Day 6 and Day 14.

No clinical sequelae were detected during a one-year follow-up.

## Discussion

PH is a rare event, with an incidence rate of 0.5–1% [22]. Despite the decrease in stapler surgery, about 100,000 surgical procedures are performed yearly worldwide. This means that a large number of patients are affected by PH-related complications. From our data, PHs can occur after any stapled procedure, regardless type of operation or technique. The initial overuse of stapling techniques by surgeons with no experience in proctologic surgery or stapler use is considered the main reason for a great number of serious complications.

Literature reviews show that only a few scientific works have focused on PH, most of them describing nonhomogeneous and isolated treatment approaches with great variability in outcomes [13, 22–24]. The main case series with reports of complications were published by surgeons who do not usually perform stapling procedures [7, 14, 20]. Further, there is little information on how to prevent or treat adverse events. The choice of aggressive surgical treatment is often guided by a panic approach [23–26]. Blouhos reported a case of a progressive PH treated with a laparotomy and an anterior rectal resection [27]. Augustin described a case of intramural rectal hematoma that was turned out using Hartmann’s operation [23]. Detruit reported a giant rectovaginal hematoma that was first drained through transanal access and subsequently using a transvaginal approach [28]. Naldini described 15 cases of PH, 10 stable and five progressive: the stable cases required spontaneous or surgical drainage, whereas the various treatment approaches for progressive cases included perineal incision and packing, rectovaginal compression with a Sengstaken–Blakemore tube, laparotomy and bilateral ligation of internal iliac arteries (resulted in a colostomy), rectal vessel ligation through laparotomic access and angiographic embolization [13]. Grau and Ertem described cases of stable hematomas that were successfully treated with a simple conservative approach involving clinical observation, peripheral nutrition and blood transfusions [29, 30].

There are other examples of conservative treatment approaches in the literature [31]. In particular, Popivanov collected a series of PHs after STARR by different surgeons and proposed a decision-making algorithm of treatment [22].

The diagnosis of PH can be challenging, and anal bleeding is not a crucial sign. In our study, eight out of 14 patients (57%) with PH did not show any bleeding, with the remaining six cases displaying minimal hematochezia. All patients complained of anal pain, and six cases were associated with both peritonism and urinary retention.

Digital rectal examination can reveal wide and soft swelling of the rectal wall. Blood tests may show an immediate, severe decrease in hemoglobin or a late increase in white cells or C-reactive protein (C-RP) levels. Contrast CT scans should be mandatory, as they enable the precise detection of the size and localization of PH.

The present case series and literature review were conducted to identify an algorithm for the treatment of PH. First, perirectal hematoma can be classified according to localization, time of onset and clinical evolution. With regard to localization, posterior hematomas usually drain spontaneously through the suture line within the first week, while anterior hematomas are more symptomatic. In women, proximity to the vagina is a risk factor for abscesses and rectovaginal fistulas, and transanal surgery may be required for early drainage.

Second, PH can be classified according to onset time: intraoperative hematomas allow immediate surgical drainage. Early postoperative hematomas are diagnosed within 24 h after the surgical procedure, usually based on persistent anal pain, signs of peritonitis with urinary retention and abdominal/pelvic pain with paralytic ileus and sudden hemoglobin decrease. Late-onset PH is rarely seen with insidious symptoms, specifically anal or pelvic discomfort/pain and fever with leukocytosis and C-RP increase.

The third fundamental classification is according to evolution: PHs may be stable or progressive and life-threatening [13]. A contrast CT scan can help detect the source of bleeding, and arteriography with embolization should be performed immediately after detection. The great majority of hematomas tend to remain stable or become stable after arteriography with embolization [13, 21]. Abdominal surgery with drainage, hemostasis and packing [25] should be attempted when these procedures fail in cases of severe, progressive PH [25]. Major surgical procedures involving colostomy [23, 26, 27, 32], demolitive resections and ligature of internal iliac vessels [12, 21] should be avoided whenever possible.

**Fig. 1:**
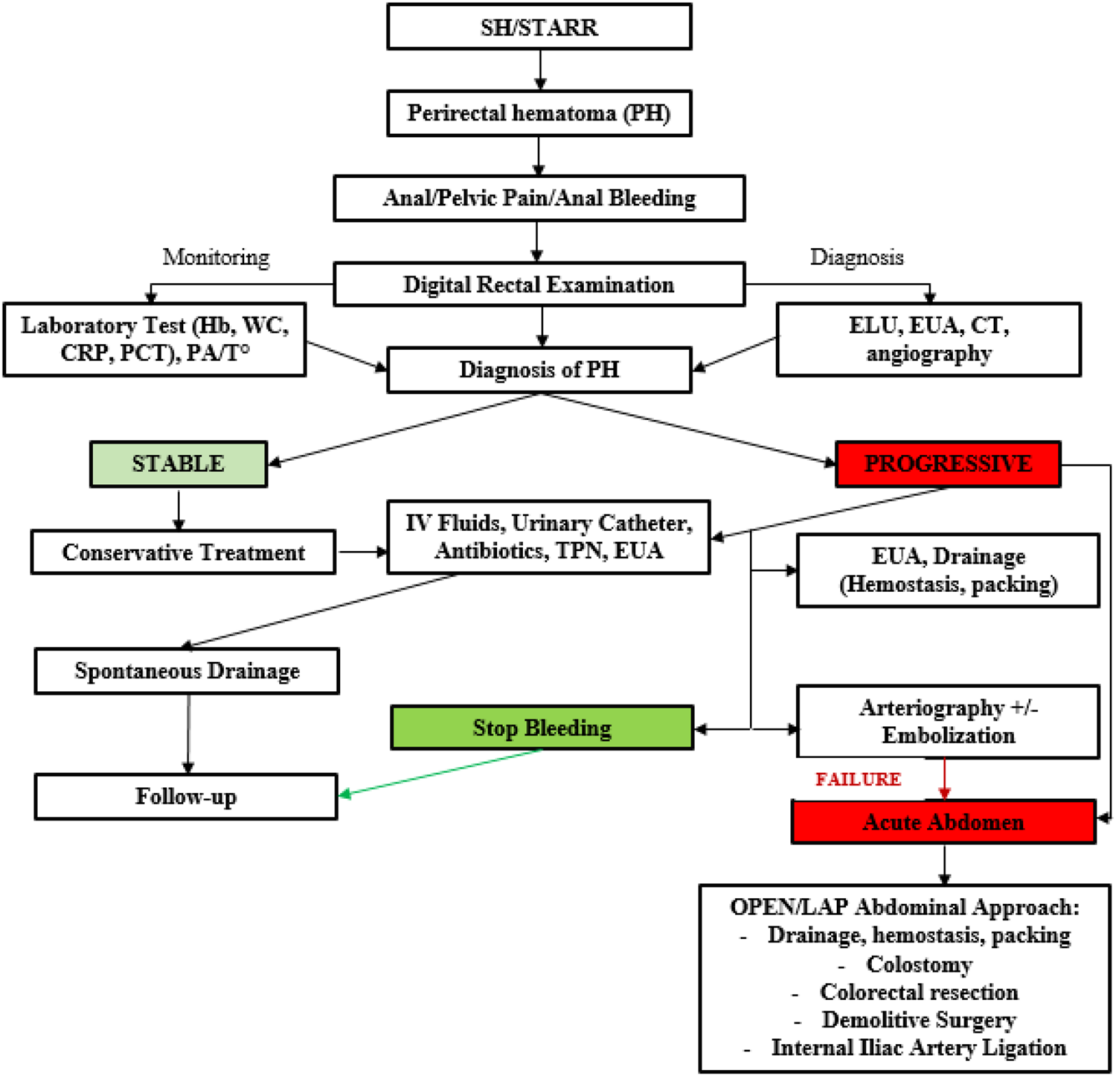
Diagnostic and therapeutic algorithm for perirectal hematoma after stapling procedures

Having analyzed the characteristics of patients with PH and the evolution of the condition, we propose a standardized management approach with a ready-to-use flowchart to minimize the risk of wrong, panic-guided, catastrophic treatment decisions (Fig. 1). In the present case series, when clinical signs, digital rectal examination and CT confirmed the presence of a hematoma, IV fluids and antibiotics (ciprofloxacin + metronidazole) were given. A bladder catheter and parenteral nutrition were added to the treatment process for patients with peritonism. Transanal, transperineal or CT-guided drainage of a stable hematoma was not effective (1/14; 7%), and spontaneous drainage was the most frequent evolution (9/14; 64%). All PHs were reabsorbed, and a long-term follow-up showed no further complications. PH cases with stable hemoglobin levels only required clinical observation and medical therapy; if hemoglobin levels were unstable and signs of active bleeding were observed, even without intrarectal bleeding, a contrast CT with arteriography was mandatory. The embolization of the source of bleeding could turn progressive hematomas into stable ones. This procedure can also be repeated when necessary.

With this approach, none of the PH patients in the present case series were referred for major abdominal surgery. This surgery should be reserved only for failed cases with persistent bleeding and signs of peritonism. The abdominal surgical plan should focus on drainage of hematoma, hemostasis and packing, and aggressive surgery should be avoided. The limitations of this study are the low number of patients with PH presentation, the inability to perform a statistical analysis of the data, and the retrospective design.

## Conclusion

Patients with hemodynamically stable PH can be treated safely and effectively without a surgical approach. These patients need to be monitored using clinical parameters, CT and blood count evaluations and treated with medical therapy and antibiotics to prevent septic complications. Most PHs are stable and evolve with spontaneous drainage or reabsorption.

In rare cases of progressive PH, angiography with embolization is crucial to avoid major surgery and the associated severe complications. Aggressive abdominal surgery should be reserved only for patients with progressive PHs upon failure of angiographic embolization.

## Data Availability

All original data and materials are available on request.

## Abbreviations

ODS: Obstructed defecation syndrome
THD: Transanal hemorrhoidal dearterialization
PH: Perirectal hematoma
STARR: Stapled transanal rectal resection
EUS: Endoluminal ultrasound
PO: Postoperative
